# A Case of Perineal Abscess

**Published:** 1882-12

**Authors:** E. Carleton

**Affiliations:** New York


### CLINICAL BUREAU.

---

### A CASE OF PERINEAL ABSCESS.

E. Carleton, M. D., New York.

*Read before the Central N. Y. Hom Medical Society, Sept. 21st, 1882.*

Mr. President and Members of the Society: Two years ago you elected me to honorary membership. To-day I offer a contribution to your archives in token of my appreciation of your partiality.

On the 30th day of October, 1877, my friend Dr. ———, a well-known alienist, called at my office and said that he wished to have me take his case in hand and *cure* him, if possible, no matter how much time might be required. He knew that the average surgeon, though attached to the homœopathic school and commonly supposed to be a homœopath, would regard his malady as a local one and rely upon the knife in treatment; he believed that my treatment would be strictly Hahnemannian, and that he desired.

Then he gave the following history: In 1864–5, he rode a great deal and was frequently galled by the saddle. Since that experience, horseback exercise has always caused a sensation of soreness in the perinæum. The 5th of last July he rode by railway a few hours; felt sore; discovered a lump, which increased in size in spite of a compress; noticed fluctuation about the 6th of August; took *Hepar* and applied poultices, which in due time caused a discharge of bland pus, moderate in quantity. Ten days before the abscess broke had a chill, and another two days after the breaking. Apparently did well the next three weeks. Then had cold, clammy extremities, and perspired freely after slight exertion. A visit to the country the first week in October seemed to do good. Besides *Hepar*, had taken *Silicea* and *Mercury* and thought the latter had helped some.

By questioning I learned further that occasionally a smarting, stinging pain had been felt—sometimes more like cutting, with itching around the sore place, which had never healed nor ceased to discharge. Question, were any of the sensations just mentioned due to the application of *Carbolic acid*, which had been employed in dressing? I thought not, for there was no aggravation after each new dressing. There were *extreme sensitiveness of parts when sitting*, a sensation of fullness in bladder, frequent urination, aching pain across sacrum. Generally felt worse in the morning, and better after exercising moderately, but worse from getting tired. Pus thin, like blood and water, or "like beef brine," scanty, unirritating. He had lost much flesh, coughed slightly, felt weak through the chest; lung history of family bad. The constitutional symptoms were fully developed before the abscess formed. He thought that overwork and irregular living had excited the attack. Æt. thirty-five. All I have mentioned in this paragraph was given in answer to *indirect questions*, as Hahnemann directs.

Having obtained the subjective symptoms, which I consider always the most important by far, I next made physical examination, as it is usually called. That is to say, I found the following objective symptoms: Superficial opening just to left of raphé; sinus thence backward, nearly in line of incision for lateral lithotomy, consequently bagging of fundus (from accumulation of pus), which was highly inflamed; from opening forward sinus extended well up into left side of scrotum, making, all told, about five inches in length that permitted the passage of an ordinary probe. No communication with the intestine could be discovered.

Comparing the materia medica, I found that *Berberis vulgaris* had the nearest resemblance of all proven drugs to the symptoms of the case. He immediately received a dose of *Berberis*$^{200}$, and blank powders to follow. No medicated application, only a compress to prevent bagging as much as possible. Hahnemannian diet and regimen.

November 10th he wrote to inform me of a severe aggravation—"gnawing, pinching pains; distressing, sharp—feels as if flesh were being eaten away; discharge thin, bloody, odorless, with occasionally a little pus mixed." Powders discontinued till he could see me. He urged me to visit him (sixty-five miles from New York). I wrote, advising him to keep quiet in bed.

November 18th saw him in bed. About two tablespoonfuls of pus discharged in twenty-four hours; less swelling and redness; sinus a little shorter. Feels better. I gave sugar.

December 14th.—Improvement stopped. Gave one dose *Berberis*$^{5,000}$, which caused aggravation, followed by amelioration.

January 21st, 1878. Improved, but these new symptoms appeared: Pupils dilated, imperfect reaction against light; bad sleep alternate nights; itching in anus; felt like cracks running into rectum; aching, corrosive pain in abscess when exposed to air; nausea in pharynx. Then I yielded to entreaty and gave a dose of *Belladonna*$^{200}$ to relieve the new symptoms—an intercurrent dose, so to speak. This was an infraction of the rule to give no medicine for new symptoms that may arise when a case is doing well under the influence of the similar remedy. Of course, retribution followed, and I was not caught that way again. *Belladonna* relieved the new symptoms, to be sure, but it was a palliation only, and the patient, knowing what it was that I had given, repeated the dose afterward as occasion seemed to require. Improvement stopped, and it became necessary to study the case anew. The character of the pain decided in favor of *Carbo. veg.*, and one dose of the 200th was given January 25th, which was followed by improvement.

Feeling now that I had the confidence of the patient sufficiently to warrant the step, I dropped the *Placebo* part of the business almost entirely. I cannot give a full history of the next two months. Sickness and death in my own family caused me to abandon my professional cares for the while. It is certain, however, that my patient took cold and had acute pharyngitis, and took for it, on his own responsibility, *Causticum* and *Phytolacca.* The acute trouble was stopped and the chronic ceased to improve. In this muddled state of affairs it seemed best to give a dose of *Sulphur* $^{200}$, which caused great improvement, followed by a stationary condition. During this period the lower end of the sinus opened, thus forming a fistula.

Under date of April 7th he wrote as follows: "I am getting along finely, but think it is about time to have another dose of medicine. I am around every day; have very little pain; eat well; sleep well, and the discharge, although rather more profuse at times, is still bland and unirritating. These are about all the *symptoms* I have, and they are not bad." I sent one dose of *Sulphur* $^{1,000,000}$ (Bœricke & Tafel). He responded to this April 13th, with—"Yours received and contents swallowed! I am doing *most excellently well,* and my spirits are almost constantly at *summer heat.* I am *beginning* to be an admirer of conservative surgery of the *pure* homœopathic variety. Am working hard all the time and seem to be *growing fat* on it, too."

May 6th. Itching—worse after bathing—was met with another dose of *Sulphur* $^{mm}$.

September 17th. Reported himself hale, hearty, fat, strong, and active. Local trouble slight.

December 27th, 1879. "I *am* fat, healthy, and happy, but the fistula has not *entirely* healed. It seems to improve gradually from month to month, although for a few weeks past I have been able to note but little improvement. So I *guess* you had better send me another powder and wait a *little longer*—say another year—before reporting the case." Accordingly I sent another dose of *Sulphur* $^{mm}$. A month later he wrote to inform me that "that last dose of medicine did me a *power of good.*" The following October he received his last dose of medicine, which was again *Sulphur* $^{mm}$. In February, 1881, we met, when he declared himself perfectly well. His health has continued good in all respects ever since.

